# Analyzing Cooking Efficiency of Gradoli Purgatory Beans: Effects of Dehulling, Malting, and Monovalent Carbonates

**DOI:** 10.3390/foods13162505

**Published:** 2024-08-09

**Authors:** Alessio Cimini, Lorenzo Morgante, Mauro Moresi

**Affiliations:** Dipartimento per l’Innovazione nei Sistemi Biologici, Agroalimentari e Forestali, Università della Tuscia, Via S. C. de Lellis, 01100 Viterbo, Italy; a.cimini@unitus.it (A.C.); lorenzo.morgante@unitus.it (L.M.)

**Keywords:** bean softening kinetics, carbon footprint of bean cooking, decorticated beans, Gradoli Purgatory beans, malted and dehulled beans, optimal cooking time, sodium or potassium carbonate/bicarbonate, texture profile analysis

## Abstract

Legumes, rich in protein, fiber, and micronutrients, are increasingly popular in pulse-based and gluten-free foods despite global consumption stagnating at 21 g/day due to taste, low protein digestibility, anti-nutrients, and long cooking times. Bean resistance to cooking causes textural defects like the hardshell and hard-to-cook phenomena. The pectin–cation–phytate hypothesis explains why soaking beans in sodium salts reduces cooking time by enhancing pectin solubility in water. Gradoli Purgatory beans (GPB), from Italy′s Latium region, were malted, reducing phytic acid by 32% and oligosaccharides by 63%. This study evaluated the hardness of cooked GPB seeds in various conditions, including decorticated or malted states, using a modified standard method. Cooking at 98 °C for 7–75 min on an induction hob with a water-to-seed ratio of 4 g/g was tested. Soaking was applied before cooking for conventional seeds only, followed by texture analysis. Conventional GPBs were adequately cooked if their cotyledons disintegrated upon pressing, requiring a force peak of 250 to 220 N and cooking times of 52 to 57 min. Malted, decorticated, and split GPBs cooked similarly to raw decorticated and split ones, with times of 32 and 25 min, respectively. Faster cooking was due to bean coat removal and splitting, not chemical changes. Sodium or potassium carbonate/bicarbonate at 1–2 g/L improved cooking efficiency, with 2 g/L of sodium carbonate reducing cooking time to 13 min. Higher concentrations caused non-uniform cooking. Cooking malted, decorticated, and split GPBs in sodium-carbonated water reduced greenhouse gas emissions from 561 to 368 g CO_2e_/kg, meeting the demand for eco-friendly and nutritionally enhanced plant protein sources.

## 1. Introduction

The global production of legumes reached approximately 102 million metric tons (Mg) in 2023 [1]. Among these, dry beans, chickpeas, and lentils accounted for around 28.35, 18.1, and 6.66 million Mg, respectively [2]. Despite the positive trend in pulse sales, particularly in foods formulated for diabetic and celiac patients, and a projected market growth with a compound annual growth rate of 1.7% between 2024 and 2032 [1], the global per capita consumption of legumes remains stagnant at around 21 g per day [3]. This might be related to the extensive time needed for meal preparation, potential digestive problems [4], and high levels of nutrient inhibitors [5]. Furthermore, raw or undercooked beans contain phytohemagglutinin, a toxic protein that can be inactivated by cooking beans for 10 min at 100 °C after soaking them for at least 5 h and discarding the soaking water [6].

Bean resistance to cooking often involves two main textural defects: hardshell (HS) and hard-to-cook (HTC). HS involves an impermeable seed coat, while HTC involves water-imbibed cotyledons that do not soften during boiling. These defects stem from prolonged storage at elevated temperatures and fluctuating relative humidity, both low and high [7]. The hydration of beans during steeping and cooking is determined by factors such as bean size, seed coat thickness, chemical makeup, and hilum and micropyle dimensions, as well as external conditions, such as temperature, pH levels, and the concentration of solids in the soaking medium.

Three primary mechanisms are thought to cause the HTC effect in stored beans: (i) the limited hydration of intracellular protein; (ii) pectin insolubilization in the middle lamella due to calcium and/or magnesium ions, following the action of pectin methyl esterase (PME) and phytase; and (iii) the cross-linking of phenolics and/or protein in the middle lamella [8,9]. Recent studies [8,10,11] support the pectin–cation–phytate hypothesis or the dual enzyme mechanism, which involves the actions of phytase and PME. During storage, phytic acid, which strongly binds to calcium and magnesium ions, is hydrolyzed by phytase, releasing phosphate, Ca^++^, and Mg^++^ into the cotyledon cells. These ions then cross-link with middle lamella pectin. Concurrently, PME hydrolyzes pectin to methanol and pectinic acid, forming insoluble calcium and magnesium pectates, leading to hard beans. Microscopic observations revealed that bean softening mainly results from the solubilization of pectin and cell wall polysaccharides, with seed coats playing a minor role [8]. Additionally, Chen et al. [11] found that during unfavorable postharvest storage condition of red haricot beans, Ca cations released during storage remained bound to cell wall pectin during soaking, whereas Mg cations leached out due to their weak binding and cell membrane damage.

Soaking beans in aqueous solutions with different sodium salts (including chloride, carbonate, bicarbonate, and phosphate) or ethylenediaminetetraacetic acid significantly decreased total cooking time by enhancing the water solubility of pectin, compared to soaking in plain water [12]. For example, Avila et al. [13] reported that using 2.5% NaHCO_3_ and 1% KCl efficiently increased the hydration level, decreases HTC in cowpeas, and reduces the time of cooking. Microscopic observations have revealed that beans soaked in sodium salt solutions exhibit improved cell separation [14]. Monovalent cations like Na^+^ and K^+^ weaken cotyledon tissue by quickly breaking pectin linkages and increasing pectin solubility [15]. Similarly, common bean cooking in 0.1 mol/L NaHCO_3_ solution accelerated softening [8]. Moreover, beans steeped in solutions containing sodium phytate reduced cooking time by chelating calcium ions from pectates, which improved cellular separation [16]. These salt treatments effectively reduced bean cooking time but slightly affected the cooked bean flavor [17]. The most effective soaking solution contained as much as 0.5% NaHCO_3_ and 2.5% K_2_CO_3_ (*w*/*v*), but the soaking water needed to be discarded and the soaked beans were washed with fresh water before cooking [17]. However, it remains uncertain whether reducing the concentration of these saline solutions might improve the final taste of cooked beans.

Among various technologies tested to reduce anti-nutrients in legumes [18,19], soaking beans to stimulate sprouting was found to enhance nutritional quality, reduce anti-nutrients, and shorten cooking time for red beans [20]. Moreover, Cimini et al. [21,22,23] showed that malting, a process commonly used in brewing, was effective for three typical varieties—Gradoli Purgatory beans, Valentano Straight Furrow chickpeas, and Onano lentils—traditionally grown in the province of Viterbo, Italy [24]. Gradoli Purgatory beans resemble Cannellini beans but have a thinner skin. Since the 17th century, they have been a traditional feature of the Purgatory Lunch in Gradoli, Italy, on the first Lensel day [25]. Rich in protein and resistant starch [22], these beans are ideal for vegetarian diets and beneficial for individuals with celiac disease or diabetes.

Longer cooking times result in higher energy use, leading to efforts to breed faster-cooking bean varieties to encourage consumption [26].

Various methods have been tested to assess cooking times, but no standardized method has been established [27]. For instance, the degree of seed penetration by the 25 plungers of an automated Mattson cooker varies from 50% to 100%, affecting cooking times. Wang and Daun [28] suggest that the nominal cooking time for pulses is based on penetrating 80% of 25 seeds pre-soaked in distilled water for 24 h at room temperature and then cooked in 1.2 L of boiling water. In contrast, the American Association of Cereal Chemists (AACC) standard method 56-36.01 [29] evaluates the firmness of a 40-g sample (pre-soaked in 160 mL of distilled water for 24 h and cooked in 1.0 L of boiling water) using a mini-Kramer shear cell compression test [29]. This method helps determine the optimal cooking time by evaluating how the firmness changes with cooking. Although this approach is more reliable due to the larger sample size, it is labor-intensive due to the difficulty of cleaning the Kramer shear cell [27]. To address this, the method was modified by increasing the cooked bean sample size from 40 to 70 g and replacing the Kramer shear cell with the Ottawa Texture Measuring System (Techlab Systems, Itasca, IL, USA), which features a square test cell with solid walls and a perforated base. Compression tests with this system generated texture profile analysis (TPA) graphs comparable to those from the Kramer shear cell [30].

Thus, this study aimed to assess the hardness of GPB seeds in various forms, including decorticated and malted, as a function of cooking time in boiling tap water or low-salt water. The evaluation utilized an adapted method to illustrate how malted decorticated beans can help reduce greenhouse gas emissions from cooking while providing nutritionally enhanced plant-based protein.

## 2. Materials and Methods

### 2.1. Raw Materials

The Gradoli Purgatory beans (*Phaseolus vulgaris*) were sourced from Il Cerqueto Srl, located in Acquapendente, Viterbo, Italy.

### 2.2. Processing

**Figure 1:**
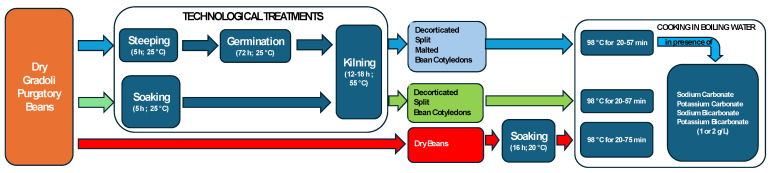
Flowchart of the production processes for malting, dehulling, and splitting the cotyledons of Gradoli Purgatory beans.

The beans either underwent a sequential process of soaking, drying, decortication, and splitting or were subjected to malting. A schematic overview of both processing methods is provided in Figure 1.

**Figure 2:**
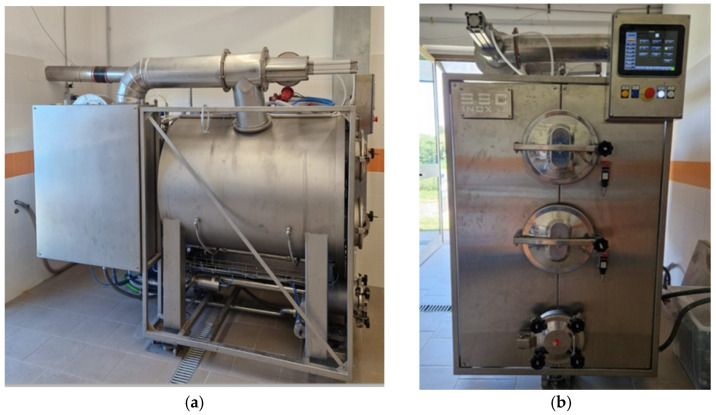
Lateral (**a**) and front (**b**) view pictures of the 100 kg pilot-scale malter used in this work.

The malting process was conducted in a 100 kg pilot-scale malting unit (Bbc Inox Srl, Possagno, TV, Italy), which automates all phases of the process (see Figure 2). The procedure involved the following steps:

(1) Washing: A total of 50 kg of dried GPBs was washed using 200 L of water and measured using an LZS-25 (D) Jectse flow meter with a measuring range of 60–600 L/h and an accuracy of 4% at the upper range.
(2) Steeping: The first phase of the malting process involved soaking the beans for 5 h in 200 L of water at 25 °C. This increased the bean water level from 12 to 54.7 ± 1.4 % (*w*/*w*) [22].
(3) Germination: After draining the steeping water, the beans were kept germinating at 25 °C with continuous humidification for 72 h. The degradation of major antinutrients, such as phytic acid and α-galactosides, was analyzed utilizing specific assay kits from Megazyme Ltd. (Bray, Ireland).
(4) Dehydration: The germinated beans were dried to a final water fraction of 10 ± 2% (*w*/*w*) by setting the maximum temperature at 55 °C for 12–18 h.

Energy consumption during the malting process was monitored using an intelligent digital AC/DC clamp multimeter model AP 570T-APP (Aoputtriver Technology Co., Ltd.).

The cotyledons of the malted GPBs were separated from the sprouted rootlets and cuticle fragments using a stone roller dehuller model D-250 (Zanotti srl, Casalvolone, Italy), which had a nominal capacity of 250 kg/h. The stone rollers gently broke the bean cuticles and rootlets without damaging the inner cotyledons. As waste materials were aspirated away, the recovery yield of split cotyledons reached 85 ± 2% of the input GPBs [22].

### 2.3. Characterization of Bean Seeds

All seeds, both before and after the malting process, were evaluated using the following physical tests: seed weight (m_S_), seed volume (V_S_), average radius (R_S_), density (ρ_S_), hydration capacity (HC), and swelling capacity (SC), as previously detailed [21].

Moisture content was measured by splitting the beans and then drying them at 110 °C for about 20 min with a Kern DAB 100-3 thermostatic scale (Kern & Sohn GmbH, Balingen, Germany). The total starch and resistant starch contents were measured using specific kits from Megazyme Ltd. (Bray, Ireland). The protein content was determined according to method 992.23 [31], using a nitrogen conversion factor of 6.25.

### 2.4. Bean Cooking Procedure

Approximately 750 g of Gradoli Purgatory bean seeds, whether whole, split, or malted, were weighed and placed into a pot. Tap water at 20 °C was added to achieve a soaking ratio of 4 g of water per g of beans [32]. The beans were soaked at this temperature for 16 h. After draining the soaking water, the hydrated beans were moved to a pot with 3 kg of water and cooked with the lid on at 98 °C for 20 to 75 min using a 2-kW induction cooker (INDU, Melchioni Spa, Milan, Italy). Decorticated and split GPB cotyledons, whether raw or malted, were cooked directly without pre-soaking. They were placed in room-temperature tap water and boiled at 98 °C for 20 to 57 min. Additionally, malted bean cotyledons were cooked without pre-soaking in tap water that contained 1 or 2 g/L of sodium (E500) or potassium (E501) carbonate (i) or bicarbonate (ii). The induction hob was first set to its maximum power of 2 kW until the water reached boiling point, then was reduced to 0.4 kW for the rest of the cooking. Keeping the pot covered throughout further minimized water evaporation. The electric energy consumed by the hob (E_S_) was recorded using a digital power meter (model MP600, RCE Srl, Salerno, Italy). Cooked beans were retrieved from the cooking water with a colander, cooled under running tap water for 90 s, and then allowed to drain. 

### 2.5. Analytical Methods

A quantity of 70.0 ± 0.5 g of cooked beans was placed into an Ottawa Texture Measuring System (Techlab Systems, Itasca, IL, USA). A 4 cm × 4 cm probe was affixed to the compression plate of a Universal Testing Machine model 3342 (Instron Int. Ltd., High Wycombe, UK), which was equipped with a 1000-N load cell to measure the compression force applied. The probe speed was set to 1 mm/s. The compression plate was initially adjusted to ensure each cooked sample was at a height of 20 mm, ensuring uniform seed orientation on the probe. After this adjustment, the plate was returned to its starting position. Following a 5-s relaxation time, each sample underwent two successive 25% compression cycles to simulate jaw action. Textural parameters were extracted from the resulting force-versus-time curves, according to Bourne [33]. The hardness of the cooked beans at 25% deformation was defined by the peak force during the first (H_1_) or second (H_2_) compression cycle. Cohesiveness, or cohesion energy resilience (CER), was calculated as the ratio of the areas under the force-versus-time curves for the second (AC_2_) and first (AC_1_) compression cycles, while the cohesion force resilience (CFR) was defined as the ratio of H_2_ to H_1_. Each texture profile analysis (TPA) test was performed at least five times.

Seed softening was modeled using a two-parameter non-linear exponential regression equation [34], given by:

H_1_ = H_10_ exp(−K_F_ t_C_) (1)

where H_1_ and H_10_ denote the current and initial peak forces during the first compression cycle, t_C_ is the cooking time, and K_F_ is the rate constant for seed softening. 

The optimal cooking time (OCT) for the beans was established by six trained panelists who evaluated the firmness of the beans at various cooking intervals. Additionally, a sensory cookability test, known as the pinching test, was performed to evaluate the softness of the beans by pressing them between the thumb and index finger. Beans were considered properly cooked if their cotyledons broke down under this pressure [35].

### 2.6. Energy Balance and GHG Emissions Associated with Bean Cooking

The theoretical energy needed to raise the initial quantities of cooking water (m_WC_) and pre-hydrated beans (m_HB_) from the initial temperature T_0_ to the boiling temperature of water (T_b_) can be calculated as follows:

E_th_ = q_sW_ + q_sHB_ (2)

with

q_sW_ = c_pW_ m_WC_ (T_b_ − T_0_) (3)

q_sHB_ = c_pHB_ m_HB_ (T_b_ − T_0_) (4)

where q_sW_ and q_sHB_ indicate the energy needed to increase the temperature of the cooking water and pre-soaked beans from the initial temperature (T_0_) to the boiling temperature (T_b_ ≈ 98 °C), and c_pW_ (=4.186 kJ kg^−1^ K^−1^) and c_pHB_ are the specific heats of the cooking water and the pre-hydrated beans, respectively. Since the pre-soaked beans had an initial moisture content of approximately 55% (*w*/*w*), the specific heat was calculated as follows:

c_pHB_ = c_pW_ × 0.55 + c_pdm_ × 0.45 = 4.186 × 0.55 + 1.5 × 0.45 = 2.977 kJ kg^−1^ K^−1^

where c_Pdm_ is the specific heat of the dry matter.

The dry matter of the beans used, which had an initial moisture content of 12% (*w*/*w*), was:

m_Pdm_ = 0.88 × 750 = 660 g

After soaking, the mass of the pre-hydrated beans (their dry matter content having reduced to 45%) was:

m_HB_ = 660/0.45 = 1466.67 g

In this case, the theoretical energy E_th_ would be:

E_th_ = 3 × 4.186 × (98 − 20) + 1466.67 × 2.977 × (98 − 20) ≈ 1320 kJ = 0.367 kWh

Given that the energy supplied by the stove (E_S_) to bring the mixture to T_b_ was 0.43 ± 0.04 kWh, the efficiency of the induction stove was approximately 85%. 

According to the Category Rules of the Product Environmental Footprint for cooking dry pasta [36], the environmental impact of bean cooking was evaluated considering only the impact category of climate change. Thus, the carbon footprint of bean cooking (CF_BC_) depends on the overall direct and indirect greenhouse gas (GHG) emissions resulting from this activity [37]. In this case, the inventory analysis included only the consumption of electricity, tap water, and salts used, as the impact resulting from the production of beans is constant for each cooking method, while the impact of disposing of cooking water was considered negligible, as observed in the case of cooking pasta [38]. Thus, CF_BC_ was estimated as follows:

CF_BC_ = m_BD_ (e_S,eff_ EF_EE_ + WPR EF_TW_ + WPR c_Si_ EF_Si_) (5)

with

e_S,eff_ = E_S_/[(1 − η_IG_) m_BD_] (6)

where e_S,eff_ indicates the specific electrical energy actually absorbed from the Italian grid to cook 1 kg of dry beans, η_IG_ (=6.1%) represents the average electrical energy grid lost in 2021 [39], WPR (=4 L/kg) is the ratio between the volume of cooking water and the quantity of dried beans to be cooked (m_BD_), c_Si_ is the concentration of the i-th salt dissolved in the cooking water; while EF_EE_, EF_TW_, and EF_Si_ are the emission factors (referring to a 100-year time horizon) associated with the production and distribution of low-voltage electrical energy in Italy, the use of tap water, and the production of the i-th salt used, respectively. Table 1 reports these emission factors, which were retrieved from the Ecoinvent database v. 3.9.1 of the LCA Simapro 9.5.0.0 software (Prè Consultants, Amersfoort, The Netherlands). In particular, by referring to the dry carbonation of potassium carbonate with a gaseous stream composed of carbon dioxide gas and steam in unitary molar ratio at 100 °C in a fluidized bed reactor, as patented by Kurtz and Winston [40], it was possible to estimate the emission factor for potassium bicarbonate using the aforementioned Simapro software, as reported in Table 1.

### 2.7. Statistical Analysis of Data

All data were reported as mean values with standard deviations. Statistical comparisons of means were conducted using Tukey′s test, with the significance level set at *p* = 0.05.

## 3. Results and Discussion

### 3.1. Physical Properties

Table 2 summarizes the key chemical and physical properties of the Gradoli Purgatory beans studied. The crude protein, total starch, phytic acid, and raffinose contents on a dry matter basis were like those found in various global bean varieties [3,41,42,43]. GPBs, with an average weight of 0.161 g per seed, are classified as very small beans [43]. Each seed had an equivalent spherical radius of 0.319 cm. Their density was comparable to certain Tunisian and Ethiopian bean varieties [44]. The swelling capacity (SC) and hydration capacity (HC) of GPBs were approximately 0.16 g/seed and 0.32 cm^3^/seed, respectively. In other dry bean genotypes [44,45], HC ranged from 0.17 to 0.54 g/seed and SC from 0.16 to 0.50 cm^3^/seed. As shown in Table 2, GPBs contained around 5.3 ± 0.3 g of raffinose and 1.15 ± 0.12 g of phytic acid per 100 g of dry mass, like common beans, which contain 0.4 to 16.1 g of α-galactosides [46] and 0.3 to 2.9 g of phytic acid [47] per 100 g of dry mass. Decorticated and split beans in their original form (DSGPB) or malted (MDSGPB) showed decreased physical properties, with HC and SC not being statistically different at a 95% confidence level. Moreover, the latter exhibited significant reductions in flatulence-inducing sugars and phytate, by about 63% and 32%, respectively, compared to the raw counterpart.

### 3.2. Bean Softening upon Cooking 

The softening of beans upon cooking was monitored using several TPA tests as a function of cooking time (t_C_). As an example, Figure S1 in the electronic supplement shows the main results of a two-cycle TPA test performed with raw GPBs, pre-soaked in tap water for 16 h and cooked for 20, 45 or 75 min. 

Table S1 in the electronic supplement shows the effect of cooking time (t_C_) on a few TPA parameters (i.e., H_1_, AC_1_, AD_1_, H_2_, AC_2_, AD_2_, CER, and CFR) of GPBs in various forms. 

**Figure 3:**
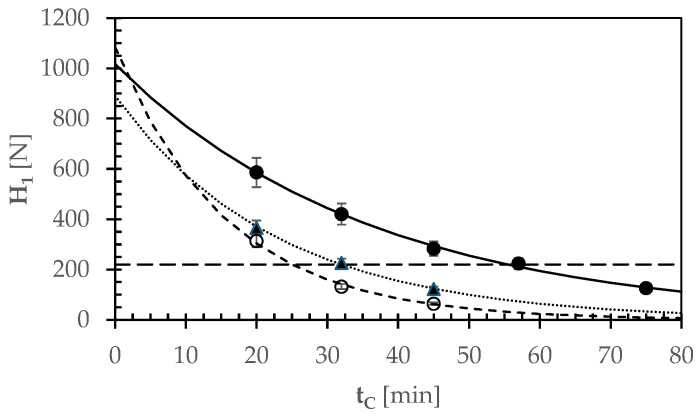
Effect of cooking time (t_C_) on the experimental hardness H_1_ of GPBs in various forms: 16 h pre-soaked (●, −); unsoaked, decorticated, and split (○, - - -); malted, decorticated and split (▲, - - -). The continuous, dotted, and broken lines were plotted using Equation (1) and the empirical coefficients listed in Table 3. The horizontal broken line represents the H_1_ value that indicates the beans are adequately cooked.

Firstly, all the textural parameters H_1_, AC_1_, AD_1_, H_2_, AC_2_, or AD_2_ exhibited an exponential decrease as t_C_ increased, as shown for instance for H_1_ in Figure 3. In contrast, the cohesion force (CFR) and energy (CER) resiliencies displayed statistically significant variations close to the optimal cooking time.

The attempt to compare the textural properties found in this study with those reported by other researchers was challenging due to variations in measurement methodologies. Different studies used various setups, such as a texture analyzer equipped with either a 25 kg_f_ [48] or 250 kg_f_ [8,14] load cell, as well as different bean varieties, preparation methods, and equipment calibrations. Additionally, the beans were positioned differently: some studies used a single half bean with the flat side of the cotyledon facing the instrument [8,48], while others compressed five beans at once [14]. Despite these differences, a consistent trend was observed across all studies, including ours: bean hardness decreases exponentially with cooking time. This trend was noted regardless of factors like average bean weight, cultivar, seed age (fresh or aged), seed coat thickness [48], and cooking water temperature [49]. These variations underscore the need for a more standardized approach to measuring and reporting the textural properties of beans to enable more accurate cross-study comparisons, as emphasized by Wood [27].

The rate of bean softening was expressed using the first peak force (H_1_) as described in Equation (1).

Table 3 presents the different least-squares empirical coefficients (H_10_, K_F_). Unsoaked, decorticated, and split GPBs exhibited a statistically significant (*p* = 0.05) greater softening rate constant (K_F_) than the malted, decorticated, and spliGPBs, likely due to the kilning process delaying the cookability of the latter. The softening rate constants assessed here ranged from 0.028 to 0.064 min^−1^, which is consistent with the range observed for different non-aged bean accessions with individual bean weights varying from 0.16 to 0.56 g/seed [48].

**Figure 4:**
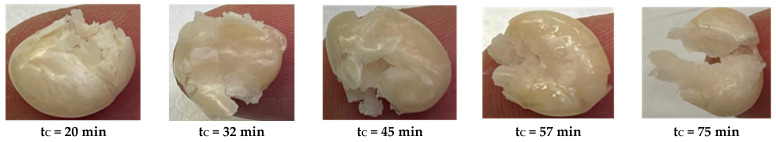
Images of pre-soaked GPBs subjected to the pinch test after cooking for 20 to 75 min.

The pinch test was not effective in precisely determining the optimal cooking time for beans. All samples cooked for more than 20 min tended to disintegrate when pressed between the thumb and index finger (Figure 4). As the cooking time (t_C_) increased from 20 to 32 min, the beans lost small pieces. For cooking times exceeding 45 min, the beans became increasingly pasty and softer when pressed, though they still retained some degree of cohesion (Figure 4).

When the trained panelists considered the Gradoli Purgatory beans (GPBs) cooked, the first peak hardness (H_1_) varied between 200 and 250 N, regardless of their pretreatments. Thus, this H_1_ range was identified as representative of optimal cooking conditions based on sensory evaluations of texture and the pinch test not only for GPBs, but also for dehulled and split GPBs (DSGPBs), or malted, decorticated, and split GPBs (MDSGPBs).

The optimal cooking times corresponding to a first peak force of around 220 N were approximately 55 min for pre-soaked GPBs, 25 min for unsoaked DSGPBs, and 32 min for MDSGPBs. Prolonged cooking times resulted in a progressive loss of texture in all beans examined. This behavior is likely associated with structural and chemical differences in the cotyledons and cuticles of the cooked beans.

It is important to note that DSGPBs were obtained by soaking, low temperature drying (as in the case of MDSGPBs), and subsequent dehulling and splitting, and were cooked without prior soaking. Despite having an initial moisture content lower than that of pre-soaked GPBs (12% vs. 55% *w*/*w*), the absence of skin and splitting into halves facilitated faster hydration and softening during cooking. 

Conversely, the production of decorticated, split, and malted Purgatory beans (MDSGPBs) involved several steps: soaking, germination, drying, dehulling to remove the outer skin and rootlets, and splitting the cotyledons. These beans were also cooked without prior soaking. Although these processing steps typically reduce cooking times compared to raw beans due to the partial degradation of cotyledons during germination, the kilning phase may have contributed to the hard-to-cook phenomenon, as indirectly indicated by the lower hydration capacity (HC) of MDSGPBs (Table 2). Additionally, the reaction of pectinic acid with calcium ions released during phytic acid degradation may have stiffened the cell walls, affecting bean texture. Despite similar physical structures, the longer optimal cooking time for MDSGPBs (32 min compared to 25 min for decorticated and split GPBs) appeared to be primarily due to changes induced by the malting process. In this context, it was not possible to confirm the reduction of cooking time for red beans due to germination, as reported by Haileslassie et al. [20], especially if the germinated beans are then preserved in dried form.

### 3.3. Effect of Monovalent Salts on Cooking Time

To reduce the optimal cooking time of malted and decorticated Gradoli Purgatory beans, they were cooked using different monovalent salt solutions. The salt concentrations used (1 or 2 g/L) were considerably lower than those tested previously [8,13,17] to avoid imparting any salted taste to the cooked beans.

Table S2 illustrates the impact of these salt solutions on the key TPA parameters of malted, decorticated, and split GPBs relative to cooking time (t_C_).

Even in this study, comparing the textural properties of the beans cooked in saltwater with those reported by other researchers [8,12,13,14,15] was challenging due to differences in measurement methodologies, average bean weights, cultivars, seed age (fresh or aged), seed coat thickness, and cooking water temperature. However, a consistent finding across studies was that adding monovalent salt carbonate/bicarbonate to the cooking water reduced cooking times. For example, de León et al. [17] found that soaking beans in a solution containing 0.5% (*w*/*v*) NaHCO_3_ and 2.5% (*w*/*v*) K_2_CO_3_ was effective, provided the soaking water was discarded and the beans were rinsed with fresh water before cooking to minimize the salty flavor. However, they did not specify the extent to which this method reduced cooking times compared to standard cooking conditions.

**Figure 5:**
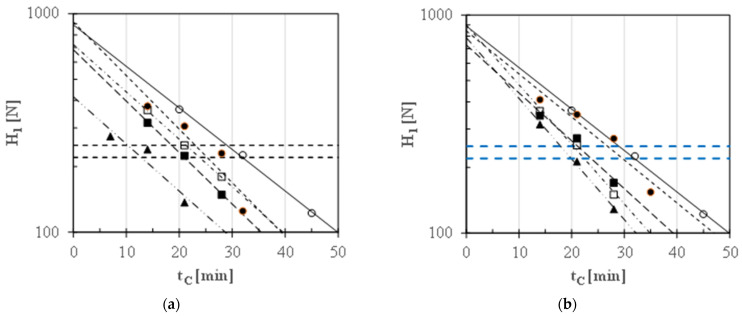
Effect of cooking time (t_C_) on the first peak hardness (H_1_) of malted, decorticated, and split GPBs cooked in tap water (○, ^____^, 0 g/L) or with different concentrations (c_Si_) of (**a**) sodium (●, - - -, 1 g/L NaHCO_3_; ☐, ^_ . _ . _^, 1 g/L Na_2_CO_3_; ■, ^_ _ _^, 2 g/L NaHCO_3_; ▲, ^_ . . _ . . _^, 2 g/L Na_2_CO_3_) or (**b**) potassium (●, - - -, 1 g/L KaHCO_3_; ☐, ^_ . _ . _^, 1 g/L K_2_CO_3_; ■, ^_ _ _^, 2 g/L KaHCO_3_; ▲, ^_ . . _ . . _^, 2 g/L K_2_CO_3_) salt solutions. The different lines were plotted using the regression equations listed in Table 3, while the horizontal broken lines indicate the range of H_1_ values representing chewable cooked beans.

Figure 5 shows the first peak hardness (H_1_) of MDSGPBs cooked in tap water or with different sodium or potassium salt concentrations versus cooking time (t_C_), where the various lines were plotted based on the least-squares regressions detailed in Table 3.

Due to the linearity of all the semilogarithmic plots, it was possible to estimate the optimal cooking time (OCT) at which malted, decorticated, and split Gradoli Purgatory beans (MDSGPB) cooked in different monovalent salt solutions exhibited the first peak hardness H_1_ of around 220 N.

**Figure 6:**
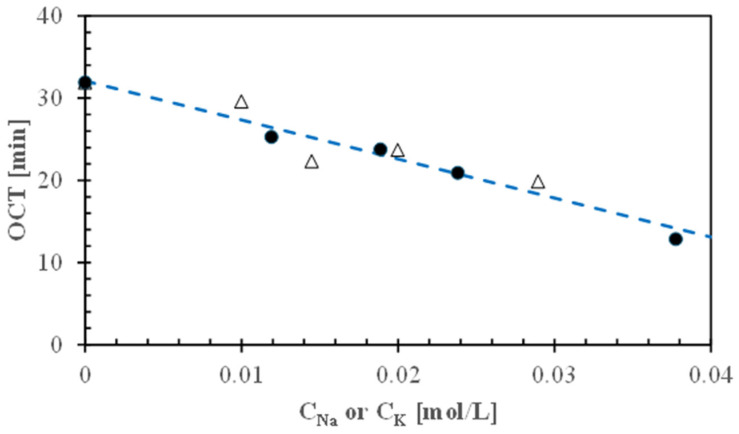
Effect of the molar concentration (C_i_) of monovalent cations (●, Na^+^; △, K^+^) in cooking water on optimal cooking time (OCT) of malted and decorticated Gradoli Purgatory beans. The broken line was plotted using Equation (7).

Figure 6 shows that these OCT values are linearly correlated to the molar concentration (C_i_) of the sodium or potassium salts used:

OCT = (32.1 ± 0.9) − (475 ± 44) C_i_     (r^2^ = 0.93) (7)

**Figure 7:**
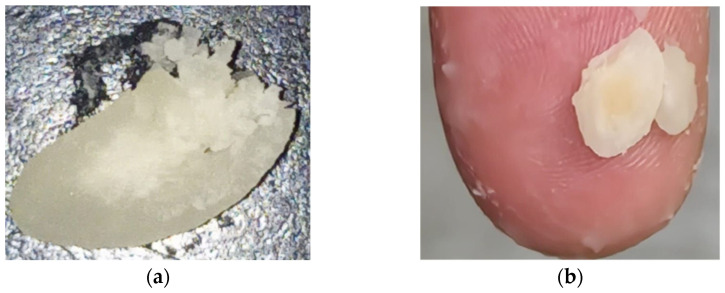
Photos of malted, decorticated, and split GPBs cooked in tap water containing 4 g/L of Na_2_CO_3_ for 15 min (**a**) and submitted to the pinch test (**b**).

An optimal cooking time as short as 12.9 min was obtained when the cooking water contained about 0.038 mol/L of Na^+^, equivalent to a sodium carbonate concentration of 2 g/L. Thus, not only were carbonates found to be more effective than bicarbonates, but sodium carbonate was also more effective than potassium carbonate. Any addition of salts at levels greater than 2 g/L caused uneven cooking. For instance, two distinct zones can be observed in malted beans cooked in tap water containing 4 g/L of Na_2_CO_3_ for 15 min, as shown in Figure 7a. The outer zone appeared well-hydrated and cooked, while the inner zone remained tough. Furthermore, Figure 7b illustrates the excessive cooking of the outer zone, which easily disintegrated upon pinching, leaving a gelatinous residue on the finger. This phenomenon also occurred in standard cooking, but the difference between the two zones was more pronounced at salt concentrations above 2 g/L compared to standard cooking.

### 3.4. Bean Cooking Energy and Carbon Footprint Estimation

**Figure 8:**
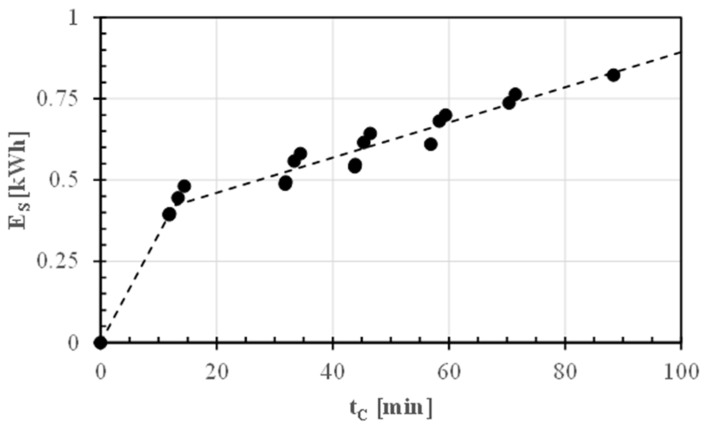
Electric energy (E_S_) consumed by the induction hob to cook about 750 g of GPBs (pre-soaked in 3 L tap water for 16 h) versus cooking time (t_C_). The broken lines were plotted using Equation (8) or Equation (9).

Figure 8 depicts the progression of electric energy (E_S_) consumed by the induction hob during the cooking of about 750 g of GPBs (pre-soaked in 3 L of tap water for 16 h) at 98 °C, with a water volume of four times the weight of the dried beans.

Therefore, the energy (E_S_) consumed by the induction stove can be predicted as follows:

E_Sc_(t) = 0.0333 t_C_          for t_C_ ≤ 12.9 ± 1.3 min (8)

E_Sc_(t) = (0.0054 ± 0.0004) t_C_ + (0.353 ± 0.018) (r^2^ = 0.93)  for t_C_ > 12.9 ± 1.3 min (9)

Considering the optimal cooking times, the electric energy required to cook 1 kg of pre-soaked raw beans would be approximately 0.87 kWh. In contrast, this energy consumption decreased to 0.65 kWh or 0.70 kWh for 1 kg of unsoaked DSGPBs or MDSGPBs, respectively.

Table 4 presents the parameters used to calculate the carbon footprint (CF_BC_) associated with cooking dry beans in water, as GPBs are either hulled and split (DSGPB) or malted, hulled, and split (MDSGPB), then cooked in water with different concentrations of sodium or potassium bicarbonate/carbonate.

As highlighted previously, the hulling phase, which allows the removal of the outer cuticle, and the splitting of bean cotyledons reduced the cooking time of Gradoli Purgatory beans by 55%, from 55 to 25 min. A similar cooking time of 32 min was also observed for malted, hulled, and split beans, clearly demonstrating the negative effect of the cuticle on cooking time. The addition of sodium or potassium salts to the cooking water further reduced the cooking time of malted, hulled, and split beans. For example, adding 2 g/L of sodium bicarbonate or carbonate reduced the optimal cooking time of Gradoli Purgatory beans to about 21 or 13 min, respectively.

The impact of the bean cooking phase was primarily due to energy consumption, as the contribution of tap water and salts was negligible (Table 4). Cooking 1 kg of dried Gradoli Purgatory beans, pre-soaked in tap water for 16 h, on an induction stove resulted in estimated GHG emissions of 561 g CO_2e_, which decreased by 25% for unsoaked hulled and split beans (420 g CO_2e_/kg) and by 34% for malted, hulled, and split beans cooked in saltwater with 2 g/L of sodium carbonate (368 g CO_2e_/kg). 

## 4. Conclusions

Germinated, dried, hulled, and split Gradoli Purgatory beans exhibited significant reductions in flatulence-inducing sugars (by 63%) and phytate (by 32%) compared to raw beans. While conventional beans required 52–57 min to reach the desired hardness, decorticated and split beans, whether as such or malted, needed only 25 or 32 min, representing a 56% or 44% reduction in cooking time, respectively, without preliminary soaking. Adding sodium or potassium carbonate/bicarbonate to the cooking water further decreased cooking times. Specifically, 0.038 mol/L Na^+^ (2 g/L Na_2_CO_3_) reduced the cooking time to 12.9 min and lowered greenhouse gas emissions to 368 kg CO_2e_ per kg of dry beans. Carbonates proved more effective than bicarbonates, with sodium carbonate being the most efficient. However, higher salt concentrations caused uneven cooking, resulting in a very soft outer zone and a hard internal core.

This study concludes that cooking time primarily depends on the bean′s cuticle structure and size, not on germination, which prolonged cooking time by releasing calcium and magnesium ions. Malting, decorticating, splitting, and cooking beans in sodium-carbonated water improved nutritional value, reduced environmental impact, and addressed barriers to legume consumption. Future research should investigate consumer acceptability and optimize processing conditions for better culinary and nutritional outcomes.

## Figures and Tables

**Table 1:** Main parameters used to calculate the theoretical energy consumed (E_th_) to heat the cooking water (m_W0_) and dried beans (m_BD_), with a known dry matter content (x_ss_), from room temperature (T_0_) to the boiling point (T_b_): average loss of the Italian electrical grid (η_IG)_ and emission factors for the production and distribution of low-voltage electrical energy in Italy (FE_EE_), tap water (FE_TW_), and monovalent salts used (FE_Si_).

Parameter	Value	Unit
c_pW_	4.186	kJ kg^−1^ K^−1^
c_pHB_	2.977	kJ kg^−1^ K^−1^
m_W0_	3	kg
m_BD_	750	g
x_ss_	0.88	g g^−1^
T_0_	20	°C
T_b_	98	°C
η_IG_	6.1	%
FE_EE_	410	g CO_2e_ kWh^−1^
FE_TW_	0.309	g CO_2e_ m^−3^
FE_S1_ (NaHCO_3_)	1.24	kg CO_2e_ kg^−1^
FE_S2_ (KHCO_3_)	2.36	kg CO_2e_ kg^−1^
FE_S3_ (Na_2_CO_3_)	0.441	kg CO_2e_ kg^−1^
FE_S4_ (K_2_CO_3_)	3.13	kg CO_2e_ kg^−1^

**Table 2:** Main chemico-physical properties of Gradoli Purgatory beans in their original form (GPB), decorticated and split in their original form (DSGPB) or malted (MDSGPB).

Chemico-Physical Property	GPB	DSGPB	MDSGPB	Unit
Raw protein	22.7 ± 1.7 ^a^	nd	23.4 ± 2.1 ^a^	g/100 g dm
Total starch	33.81 ± 1.66 ^a^	nd	34.96 ± 0.19 ^a^	g/100 g dm
Resistant starch	23.59 ± 0.34 ^a^	nd	22.01 ± 1.82 ^a^	g/100 g dm
Phytic acid	1.15 ± 0.12 ^a^	nd	0.78 ± 0.13 ^a^	g/100 g dm
Raffinose	5.31 ± 0.28 ^a^	nd	1.95 ± 0.20 ^b^	g/100 g dm
Seed weight (m_S_)	0.161 ± 0.006 ^a^	0.142 ± 0.008 ^b^	0.133 ± 0.002 ^b^	g/seed
Seed volume (V_S_)	0.137 ± 0.006 ^a^	0.120 ± 0.010 ^a,b^	0.103 ± 0.000 ^b^	cm^3^/seed
Mean seed radius (R_S_)	0.319 ± 0.005 ^a^	0.306 ± 0.009 ^a^	0.290 ± 0.000 ^b^	cm/seed
Seed density (ρ_S_)	1.175 ± 0.024 ^a^	1.181 ± 0.030 ^a^	1.096 ± 0.030 ^b^	g/cm^3^
Hydration capacity (HC)	0.158 ± 0.007 ^a^	0.126 ± 0.008 ^b^	0.136 ± 0.006 ^b^	g/seed
Swelling capacity (SC)	0.320 ± 0.0102 ^a^	0.253 ± 0.012 ^b^	0.263 ± 0.006 ^b^	cm^3^/seed

In each row, values with the same letter have no significant difference at *p <* 0.05.

**Table 3:** Empirical least-squares softening coefficients of differently pre-treated GPBs cooked in tap water or salt solutions at different concentrations (c_Si_) together with the corresponding coefficients of determinations (r^2^).

Bean Type	Salt	c_si_	H_10_	K_F_	r^2^
		[g/L]	[N]	[min^−1^]	
GPBs pre-soaked in water for 16 h	-	0	exp (6.92 ± 0.05) ^a^	0.028 ± 0.001 ^c^	0.996
Unsoaked, decorticated, and split GPBs	-	0	exp (6.99 ± 0.15) ^a^	0.064 ± 0.005 ^a^	0.995
Unsoaked, decorticated, split and malted GPBs	-	0	exp (6.79 ± 0.07) ^a^	0.044 ± 0.002 ^b^	0.998
	NaHCO_3_	1	exp (6.82 ± 0.38) ^a^	0.056 ± 0.015 ^a,b^	0.870
	NaHCO_3_	2	exp (6.58 ± 0.06) ^b^	0.054 ± 0.003 ^b^	0.998
	Na_2_CO_3_	1	exp (6.58 ± 0.04) ^b^	0.050 ± 0.002 ^b^	0.999
	Na_2_CO_3_	2	exp (6.03 ± 0.26) ^c^	0.050 ± 0.017 ^b^	0.892
	KHCO_3_	1	exp (6.74 ± 0.24) ^a,b^	0.045 ± 0.009 ^b^	0.920
	KHCO_3_	2	exp (6.59 ± 0.21) ^b^	0.051 ± 0.010 ^b^	0.965
	K_2_CO_3_	1	exp (6.80 ± 0.14) ^a^	0.063 ± 0.007 ^a^	0.989
	K_2_CO_3_	2	exp (6.67 ± 0.10) ^b^	0.064 ± 0.005 ^a^	0.995

In each column, values with the same letter have no significant difference at *p <* 0.05.

**Table 4:** Parameters used to calculate the carbon footprint (CF_BC_) associated with cooking 1 kg of dried beans in water, as they (GPB) are either hulled and split (DSGPB) or malted, decorticated, and split (MDSGPB), then cooked in water with different concentrations of sodium or potassium bicarbonate/carbonate.

Sample	Salt	c_Si_	t_C_	e_S,eff_	WPR	m_WS_	m_WC_	m_Si_	CF_BC,EE_	CF_BC,TW_	CF_BC,Si_	CF_BC_	e_Ri_
		[g/L]	[min]	[kWh/kg]	[L/kg]	[kg]	[kg]	[g]	[kg CO_2e_/kg]				[%]
GPB	-	0	55	0.92	4	4	4	0	0.559	0.0025	0	0.561	0
DSGPB	-	0	25	0.69	4	-	4	0	0.419	0.0012	0	0.420	−25
MDSGPB	-	0	31.9	0.75	4	-	4	0	0.451	0.0012	0	0.452	−19
	NaHCO_3_	1	25.3	0.70	4	-	4	4	0.421	0.0012	0.005	0.427	−24
	NaHCO_3_	2	20.9	0.66	4	-	4	8	0.400	0.0012	0.010	0.411	−27
	Na_2_CO_3_	1	23.8	0.68	4	-	4	4	0.414	0.0012	0.002	0.417	−26
	Na_2_CO_3_	2	12.9	0.60	4	-	4	8	0.363	0.0012	0.004	0.368	−34
	KHCO_3_	1	29.6	0.73	4	-	4	4	0.441	0.0012	0.009	0.451	−20
	KHCO_3_	2	23.7	0.68	4	-	4	8	0.413	0.0012	0.019	0.433	−23
	K_2_CO_3_	1	22.4	0.67	4	-	4	4	0.407	0.0012	0.013	0.421	−25
	K_2_CO_3_	2	19.9	0.65	4	-	4	8	0.395	0.0012	0.025	0.422	−25

## Data Availability

The original contributions presented in the study are included in the article and Supplementary Materials; further inquiries can be directed to the corresponding author.

## Supplementary Materials

The following supporting information can be downloaded at: https://www.mdpi.com/article/10.3390/foods13162505/s1. Figure S1: TPA testing of pre-soaked GPBs; Table S1: Effect of cooking time on the main TPA parameters when using GPBs in various forms; Table S2: Effect of cooking time on the main TPA parameters when cooking unsoaked MDSGPBs in variously salted tap water.

## Nomenclature

AC_1_	Force-vs.-time area during the first compression cycle [mJ]
AC_2_	Force-vs.-time area during the second compression cycle [mJ]
AD_1_	Force-vs.-time area during the first decompression cycle [mJ]
AD_2_	Force-vs.-time area during the second decompression cycle [mJ]
CER	Cohesion energy resilience (=AC_2_/AC_1_) [dimensionless]
CF_BC_	Carbon footprint of bean cooking [kg CO_2e_/kg]
CF_BC,EE_	Contribution of the electric energy to CF_BC_ [kg CO_2e_/kg]
CF_BC_,_Si_	Contribution of the i-th salt to CF_BC_ [kg CO_2e_/kg]
CF_BC_,_TW_	Contribution of tap water to CF_BC_ [kg CO_2e_/kg]
CFR	Cohesion force resilience (=H_2_/H_1_) [dimensionless]
C_i_	Molar concentration of the i-th monovalent cation [mol/L]
c_Pdm_	Specific heat of the dry matter [kJ kg^−1^ K^−1^]
c_pHB_	Specific heats of pre-hydrated beans [kJ kg^−1^ K^−1^]
c_pW_	Specific heats of cooking water [kJ kg^−1^ K^−1^]
c_Si_	Concentration of the i-th salt dissolved in the cooking water [g/L]
DSGPB	Decorticated and split Gradoli Purgatory beans
E500i	Sodium carbonate
E500ii	Sodium bicarbonate
E501i	Potassium carbonate
E501ii	Potassium bicarbonate
EF_EE_	Emission factor associated with the Italian production and distribution of low-voltage electrical energy [g CO_2e_/kWh]
EF_Si_	Emission factor associated with the production of the i-th salt used [g CO_2e_/kg]
EF_TW_	Emission factor associated with the use of tap water [g CO_2e_/m^3^]
E_S_	Electric energy supplied by the induction hob [kWh]
E_Sc_	Estimated energy supplied by the induction stove using Equation (8) or Equation (9) [kWh]
e_S,eff_	Specific electrical energy absorbed from the Italian grid for bean cooking [kWh/kg]
E_th_	Theoretical energy needed to heat the water-bean mixture [kWh]
F	Compression force [F]
GHG	Greenhouse gas
GPB	Gradoli Purgatory beans
H_1_	Force peak on the first 25% compression cycle [N]
H_10_	Initial peak forces on the first compression cycle [N]
H_2_	Force peak on the second 25% compression cycle [N]
HC	Hydration capacity [g/seed]
HS	Hardshell
HTC	Hard-to-cook
K	Rate constant of seed softening [min^−1^]
m_BD_	Mass of dry beans to be cooked [kg]
MDSGPB	Malted, decorticated, and split Gradoli Purgatory beans
m_HB_	Mass of pre-hydrated beans [kg]
m_Pdm_	Dry matter in beans [kg]
m_S_	Seed weight [g/seed]
m_Si_	Mass of the i-th salt added [kg]
m_WC_	Mass of cooking water [kg]
m_WS_	Mass of water used during bean steeping [kg]
OCT	Optimum cooking time [min]
*p*	Probability level [dimensionless]
PME	Pectin methyl esterase
q_sHB_	Energy required to raise the temperature of pre-soaked beans [kJ]
q_sW_	Energy required to raise the temperature of cooking water [kJ]
r^2^	Coefficient of determination
R_S_	Mean radius of beans [cm]
SC	Swelling capacity [cm^3^/seed]
t	Compression time [s]
t_C_	Cooking time [min]
T_0_	Initial temperature of cooking water and beans [°C]
T_b_	Boiling temperature of water [°C]
TPA	Texture profile analysis.
V_S_	Seed volume [cm^3^/seed]
WPR	Cooking water-to-dry bean ratio [L/kg]
x_SS_	Dry matter content of raw beans [g/g]
**Greek symbols**	
ρ_S_	Seed density [g/cm^3^]
ε_Ri_	Percentage reduction of the generic i-th CF_BC_ compared to the reference CF_BC_ value [%]
η_IG_	Average electrical energy grid lost [%]

## References

[1] Expert Market Research (2023). Global Pulses Market Report and Forecast 2024–2032. https://www.expertmarketresearch.com/reports/pulses-market.

[2] FAO (2023). Bean Production—FAO [Dataset]. Food and Agriculture Organization of the United Nations, Production: Crops and Livestock Products [Original Data]..

[3] Rawal V., Navarro D.K. (2019). The Global Economy of Pulses.

[4] de Almeida Costa G.E., Da Silva Queiroz-Monici K., Pissini Machado Reis S.M., De Oliveira A.C. (2006). Chemical composition, dietary fibre and resistant starch contents of raw and cooked pea, common bean, chickpea and lentil legumes. Food Chem..

[5] Gebrelibanos M., Tesfaye D., Raghavendra Y., Sintayeyu B. (2013). Nutritional and health implications of legumes. Int. J. Pharm. Sci. Res..

[6] FDA (Food and Drug Administration) (2012). Phytohaemagglutinin (Kidney Bean Lectin). Bad Bug Book, Foodborne Pathogenic Microorganisms and Natural Toxins.

[7] Wainaina I., Lugumira R., Wafula E., Kyomugasho C., Sila D., Hendrickx D. (2022). Insight into pectin-cation-phytate theory of hardening in common bean varieties with different sensitivities to hard-to-cook. Food Res. Int..

[8] Chigwedere C.M., Olaoye T.F., Kyomugasho C., Kermani Z.J., Pallares Pallares A., Van Loey A.M., Grauwet T., Marc E., Hendrickx M.E. (2018). Mechanistic insight into softening of Canadian wonder common beans (*Phaseolus vulgaris*) during cooking. Food Res. Int..

[9] Mubaiwa J., Fogliano V., Chidewe C., Linnemann A.R. (2017). Hard-to-cook phenomenon in bambara groundnut (*Vigna subterranea* (L.) Verdc.) processing: Options to improve its role in providing food security. Food Rev. Int..

[10] Chen D., Bernaerts T., Stephane Debon S., Oduah C.O., Zhu L., Wallecan J., Hendrickx M., Kyomugasho C. (2023). Novel insights into the role of the pectin-cation-phytate mechanism in ageing induced cooking texture changes of Red haricot beans through a texture-based classification and in situ cell wall associated mineral quantification. Food Res. Int..

[11] Chen D., Ding A., Zhu L., Grauwet T., Van Loey A., Hendrickx M., Kyomugasho C. (2023). Phytate and mineral profile evolutions to explain the textural hardening of common beans (*Phaseolus vulgaris* L.) during postharvest storage and soaking: Insights obtained through a texture-based classification approach. Food Chem..

[12] Perera D., Devkota L., Garnier G., Panozzo J., Dhital S. (2023). Hard-to-cook phenomenon in common legumes: Chemistry, mechanisms and utilization. Food Chem..

[13] Avila B.P., Santos dos Santos M., Nicoletti A.M., Alves G.D., Elias M.C., Monks J., Gularte M. (2015). AImpact of different salts in soaking water on the cooking time, texture and physical parameters of cowpeas. Plant Foods Hum. Nutr..

[14] Alpos M., Leong S.Y., Liesaputra V., Oey I. (2022). Influence of pulsed electric fields (PEF) with calcium addition on the texture profile of cooked black beans (*Phaseolus vulgaris*) and their particle breakdown during in vivo oral processing. Innov. Food Sci. Emerg. Technol..

[15] Yousif A.M., Kato J., Deeth H.C. (2007). Effect of storage on the biochemical structure and processing quality of Adzuki bean (*Vigna angularis*). Food Rev. Int..

[16] Luo Y., Xie W., Luo F. (2012). Effect of several germination treatments on phosphatases activities and degradation of phytate in faba bean (*Vicia faba* L.) and azuki bean (*Vigna angularis* L.). J. Food Sci..

[17] de León L.F., Elías L.G., Bressani R. (1992). Effect of salt solutions on the cooking time, nutritional and sensory characteristics of common beans (*Phaseolus vulgaris*). Food Res. Int..

[18] Das G., Sharma A., Sarkar P.K. (2022). Conventional and emerging processing techniques for the post-harvest reduction of antinutrients in edible legumes. Appl. Food Res..

[19] Sharma N., Sahu J.K., Joshi S., Khubber S., Bansal V., Bhardwaj A., Bangar S.P., Bal L. (2022). M Modulation of lentil antinutritional properties using non-thermal mediated processing techniques—A review. J. Food Compos. Anal..

[20] Haileslassie H.A., Henry C.J., Tyler R.T. (2019). Impact of pre-treatment (soaking or germination) on nutrient and anti-nutrient contents, cooking time and acceptability of cooked red dry bean (*Phaseolus vulgaris* L.) and chickpea (*Cicer arietinum* L.) grown in Ethiopia. Int. J. Food Sci. Technol..

[21] Cimini A., Poliziani A., Moresi M. (2021). Effect of temperature on the hydration kinetics of chickpea (*Cicer arietinum* L.) and yellow soybean (*Glycine max*). Chem. Eng. Trans..

[22] Cimini A., Poliziani A., Morgante L., Moresi M. (2023). Assessment of the malting process of Purgatory bean and Solco Dritto chickpea seeds. Foods.

[23] Cimini A., Poliziani A., Morgante L., Moresi M. (2024). Antinutrient removal in yellow lentils by malting. J. Sci. Food Agric..

[24] Di Giovannantonio C., Catta M., Pica G., Casadei G. (2019). Lazio Patrimonio Agroalimentare tra Biodiversità e Tradizione.

[25] Slow Food Foundation Purgatory Beans. https://www.fondazioneslowfood.com/en/ark-of-taste-slow-food/purgatory-beans/.

[26] Bassett A., Hooper S., Cichy K. (2021). Genetic variability of cooking time in dry beans (*Phaseolus vulgaris* L.) related to seed coat thickness and the cotyledon cell wall. Food Res. Int..

[27] Wood J.A. (2017). Evaluation of cooking time in pulses: A review. Cereal Chem..

[28] Wang N., Daun J.K. (2005). Determination of cooking times of pulses using an automated Mattson cooker apparatus. J. Sci. Food Agric..

[29] Wang N., Panozzo J.F., Wood J., Malcolmson L.J., Arganosa G.C., Baik B.-K., Driedger D., Han J. (2012). AACCI approved methods Technical Committee report: Collaborative study on a method for determining firmness of cooked pulses (AACC International Method 56-36.01). Cereal Foods World.

[30] Cimini A., Poliziani A., Morgante L., Moresi M. (2023). Cooking and nutritional characteristics of malted chickpeas. Chem. Eng. Trans..

[31] AOAC (1998). Crude Protein in Cereal Grains and Oilseeds. Generic Combustion Method. AOAC Method 992.23-1992.

[32] Kajumba P.K., Okello D., Nyeinga K., Nydal O.J. (2022). Assessment of the energy needs for cooking local food in Uganda: A strategy for sizing thermal energy storage with cooker system. Energy Sustain. Dev..

[33] Bourne M.C. (2002). Food Texture and Viscosity: Concept and Measurement.

[34] Kwofie E.M., Mba O.I., Ngadi M. (2020). Classification, force deformation characteristics and cooking kinetics of common beans. Processes.

[35] Kinyanjui P.K., Njoroge D.M., Makokha A.O., Christiaens S., Ndaka D.S., Hendrickx M. (2015). Hydration properties and texture fingerprints of easy- and hard-to-cook bean varieties. Food Sci. Nutr..

[36] UNAFPA (Unions de Associations de Fabricants de Pâtes Alimentaires) (2018). Product Environmental Footprint Category Rules (PEFCR) for Dry Pasta. Vers. 3. https://docslib.org/doc/6636089/product-environmental-footprint-category-rules-for-dry-pasta.

[37] BSI (2011). Publicly Available Specification (PAS 2050) for the Assessment of the Life Cycle Greenhouse Gas Emission of Goods and Services.

[38] Cimini A., Cibelli M., Moresi M. (2019). Reducing the cooking water-to-dried pasta ratio and environmental impact of pasta cooking. J. Sci. Food Agric..

[39] TERNA Driving Energy (2022). Bilancio dell’energia Elettrica in Italia. Dati Generali 2022.

[40] Kurtz A.D., Winston A.E. (1990). Process for the Production of Potassium Bicarbonate. US Patent.

[41] Basso Los F.G., Ferreira Zielinski A.A., Wojeicchowski J.P., Nogueira A., Mottin Demiate I. (2018). Beans (*Phaseolus vulgaris* L.): Whole seeds with complex chemical composition. Curr. Opin. Food Sci..

[42] Cappa C., James D., Kelly J.D., Nga P.K.W. (2018). Seed characteristics and physicochemical properties of powders of 25 edible dry bean varieties. Food Chem..

[43] de Barros M., Prudencio S.H. (2016). Physical and chemical characteristics of common bean varieties. Ciências Agrárias Londrina.

[44] Nciri N., El Mhamdi F., Ben Ismail H.B., Ben Mansour A., Fennira F. (2014). Physical properties of three white bean varieties (*Phaseolus vulgaris* L.) grown in Tunisia. J. Appl. Sci. Agric..

[45] Ercan R., Atli A., Köksel H., Dağ A. (1994). Cooking quality and composition of dry beans grown in Turkey. GIDA.

[46] Pedrosa M.M., Guillamón E., Arribas C. (2021). Autoclaved and extruded legumes as a source of bioactive phytochemicals: A review. Foods.

[47] Sparvoli F., Bollini R., Cominelli E., Ron A.M.D. (2015). Nutritional value. Grain Legumes.

[48] Tafiire H., Njoki Wainaina I., Lugumira R., An N.T.H., Ogwok P., Grauwet T., Hendrickx M.E. (2024). A detailed study on the cooking kinetics of fresh and hard to cook common beans (Phaseolus vulgaris L.): A case study on bean accessions of different market classes. J. Food Eng..

[49] Kyomugasho C., Wainaina I., Grauwet T., Van Loey A., Hendrickx M.E. (2023). Bean softening during hydrothermal processing is greatly limited by pectin solubilization rather than protein denaturation or starch gelatinization. Food Res. Int..

