# Supplementary material for: Analyzing Cooking Efficiency of Gradoli Purgatory Beans: Effects of Dehulling, Malting, and Monovalent Carbonates
Source: Foods. 2024 Aug 9;13(16):2505. doi: 10.3390/foods13162505 (PMC11354054; doi:10.3390/foods13162505)
Supplement: Supplementary file 1 [file foods-13-02505-s001.zip › foods-3132806-supplementary.pdf]

Analyzing Cooking Efficiency of *Gradoli Purgatory* Beans: Effects of Dehulling, Malting, and Monovalent Carbonates

Alessio Cimini (a.cimini@unitus.it), Lorenzo Morgante (lorenzo.morgante@unitus.it), and Mauro Moresi (mmoresi@unitus.it)
Dipartimento per l'Innovazione nei sistemi Biologici, Agroalimentari e Forestali, Università della Tuscia, Via S. C. de Lellis, 01100 Viterbo, Italy

SUPPLEMENTARY MATERIAL

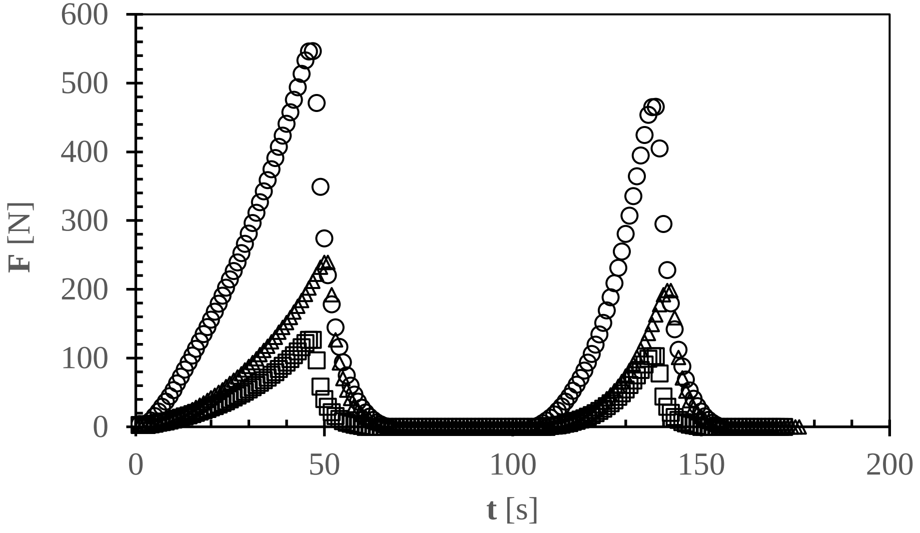

Figure S1 TPA testing using presoaked GPBs as cooked for 20 (○), 45 (△) or 75 min (□): Compression force (F) vs. compression time (t).

Table S1: Effect of cooking time (t_C) on the main TPA parameters (H_1, AC_1, AD_1, H_2, AC_2, AD_2, CER, CFR) when using GPBs in various forms: as such, presoaked in water for 16 h; unsoaked, decorticated, and split; or unsoaked, malted, and decorticated.

t_C [min]	H_1 [N]	A_{C1} [mJ]	A_{D1} [mJ]	H_2 [N]	A_{C2} [mJ]	A_{D1} [mJ]	CER [-]	CFR [-]
GPB presoaked in water for 16 h								
20	586±57 a	1649±221 a	432±76 a	521±61 a	789±106 a	345±63 a	0.263±0.011 a	0.854±0.005 a
32	420±13 b	1053±119 b	295±71 b	394±70 b	586±117 b	235±60 b	0.245±0.016 a	0.856±0.007 a
45	283±68 d	889±273 c	195±65 c	279±70 c,d	415±122 b,c	150±51 c,d	0.218±0.009 b	0.838±0.006 c
57	224±35 e	580±95 d	112±24 d	182±30 e	260±48 c	83±19 e	0.193±0.012 c	0.815±0.014 d
75	126±1 f	306±12 e	51.7±0.1 e	102.1±0.6 f	133±4 d	36.8±0.5 f	0.174±0.006 d	0.813±0.003 d
Unsoaked, decorticated, and split GPBs								
20	313±40 d	700±101 c	169±59 c	260±35 d	332±52 c	128±30 d	0.246±0.015 a	0.829±0.011 c
32	132±8 f	309±25 e	56±15 e	106±7 f	122±9 e	39±3 f	0.186±0.004 c	0.800±0.003 e
45	64±4 g	153±13 g	23±3 f	50±4 g	52±2 f	16±1 g	0.154±0.005 e	0.792±0.005 f
Unsoaked, malted, and decorticated GPBs								
20	365±20 c	819±105 c	221±17 c	309±16 c	394±32 b,c	177±12 c	0.272±0.017 a	0.847±0.006 b
32	225±19 e	507±54 d	115±13 d	187±15 e	223±22 c	88±10 e	0.228±0.006 b	0.832±0.006 c
45	122±14 f	283±28 f	52±5 e	98±12 f	110±13 e	37±4 f	0.189±0.008 c	0.798±0.013 e

In each column, values with the same Latin letter have no significant difference at $p < 0.05$.

Table S2: Cooking tests for unsoaked, malted, decorticated and split GPBs: Effect of cooking water salting with Na or K bicarbonate/carbonate at different concentrations (c_S) on the main TPA parameters (H_1, A_{C1}, A_{D1}, H_2, A_{C2}, A_{D2}, CER, CFR) *versus* cooking time (t_C).

**Salt added**	c_S [g/L]	t_C [min]	H_1 [N]	A_{C1} [mJ]	A_{D1} [mJ]	H_2 [N]	A_{C2} [mJ]	A_{D1} [mJ]	**CER** [-]	**CFR** [-]
$NaHCO_3$	1	14	377±22 [a,b]	786±65 [a]	225±16 [b]	325±18 [a,b]	415±32 [a, b]	184±12 [b]	0.29±0.01 [a]	0.86±0.01 [a]
		21	305±27 [d]	669±70 [b,c]	166±15 [d, e]	256±23 [c,d]	313±33 [d, e]	130±12 [d, e]	0.25±0.02 [a]	0.84±0.01 [b, c]
		28	229±16 [f]	521±44 [e,f]	113±9 [f]	189±14 [f]	218±19 [g]	85±7 [g]	0.217±0.003 [a]	0.823±0.009 [d, e]
		32	125±19 [j]	291±49 [j]	51±6 [i]	100±17 [h]	108±16 [k, l]	36±5 [k, l]	0.18±0.01 [a]	0.80±0.01 [f, g]
$NaHCO_3$	2	14	317±55 [c, d]	658±116 [b,c]	177±40 [c, d, e]	270±49 [c,d]	327±70 [c, d, e]	142±34 [d, e]	0.27±0.02 [a]	0.85±0.01 [b]
		21	224±43 [f, g]	505±91 [e,f,g]	105±29 [f, g]	185±37 [f]	206±52 [g, h]	78±25 [g, h, i]	0.21±0.02 [a]	0.82±0.02 [d, e]
		28	148±6 [i]	329±33 [i, j]	61±1 [i]	119±4 [g]	123±4 [j]	43.3±0.4 [k]	0.189±0.014 [a]	0.801±0.003 [f]
Na_2CO_3	1	14	361±21 [b, c]	773±33 [a]	213±16 [b, c]	308±20 [b]	389±27 [b, c]	171±15 [b,c]	0.28±0.01 [a]	0.85±0.01 [b]
		21	250±26 [e, f]	536±64 [e, f]	123±13 [f]	208±23 [e,f]	240±30 [f, g]	94±11 [g]	0.23±0.01 [a]	0.83±0.01 [c, d]
		28	179±26 [g, h]	413±53 [g, h]	78±13 [g, h]	145±22 [e]	159±26 [h, i]	57±11 [i, j]	0.19±0.01 [a]	0.81±0.01 [e, f]
Na_2CO_3	2	7	275±13 [d]	574±24 [d, e]	150±9 [e]	238±14 [d]	296±20 [e]	122±9 [e]	0.263±0.004 [a]	0.866±0.009 [a]
		14	239±9 [e, f]	503±26 [f]	122±9 [f]	201±9 [e,f]	236±19 [f, g]	95±8 [g]	0.25±0.01 [a]	0.84±0.01 [b, c]
		21	137±17 [i, j]	294±37 [j]	53±11 [i, j]	108±14 [g, h]	106±20 [k, l]	36±9 [k, l]	0.18±0.01 [a]	0.790±0.005 [g]
$KHCO_3$	1	14	409±26 [a]	845±105 [a]	252±19 [a]	349±20 [a]	453±38 [a]	204±14 [a]	0.30±0.2 [a]	0.85±0.02 [a]
		21	349±35 [b, c]	733±63 [a, b]	197±30 [c, d]	296±33 [b,c]	365±49 [c, d]	156±26 [c,d]	0.27±0.02 [a]	0.85±0.01 [b]
		28	271±33 [d, e]	602±64 [c,d]	140±25 [e,f]	224±29 [d]	269±42 [e, f]	106±22 [e,f, g]	0.23±0.02 [a]	0.82±0.01 [d, e]
		35	154±18 [h, i]	363±43 [h, i]	64±12 [h, i]	124±15 [e]	135±22 [i, j]	45±10 [j, k]	0.18±0.01 [a]	0.80±0.01 [f, g]
$KHCO_3$	2	14	346±16 [c]	723±48 [b]	202±14 [c]	296±12 [b,c]	367±26 [c]	163±11 [c]	0.280±0.003 [a]	0.855±0.006 [a]
		21	272±7 [a,b]	603±34 [c,d]	138±5 [e]	228±5 [d]	263±13 [f]	107±4 [f]	0.231±0.003 [a]	0.837±0.001 [c]
		28	170±10 [d]	399±35 [h]	72±5 [h]	139±8 [e]	149±11 [i]	52±4 [i, j]	0.185±0.006 [a]	0.814±0.003 [e]
K_2CO_3	1	14	363±11 [f]	764±12 [a]	203±11 [c]	308±10 [b]	377±12 [c]	162±9 [c]	0.268±0.009 [a]	0.849±0.002 [b]
		21	253±7 [j]	574±40 [d, e]	122±4 [f]	208±8 [e]	240±8 [f]	92±4 [g]	0.22±0.01 [a]	0.82±0.01 [d, e]

		28	150±6	c, d	318±39	i, j	56±12	i, j	123±4	e	120±14	j, k	39±11	k, l	0.190±0.002	a	0.819±0.004	d, e
K_2CO_3	2	14	315±9	f, g	633±47	c,d	176±7	d	271±8	c	319±8	d	143±6	d	0.28±0.02	a	0.86±0.01	a
		21	213±7	i	471±19	f, g	95±3	g	176±7	f	188±6	h	71±4	h	0.21±0.01	a	0.83±0.01	c, d
		28	129±9	b, c	292±27	j	44±3	j	102±8	h	96±9	l	29±3	l	0.16±0.01	a	0.79±0.01	g

In each column, values with the same Latin letter have no significant difference at $p < 0.05$.
